# Quantitative and single nucleotide RNA m^6^A detection technology boosts clinical research based on tissue and cell free RNA modification

**DOI:** 10.1002/ctm2.1082

**Published:** 2022-10-20

**Authors:** Lulu Hu

**Affiliations:** ^1^ Shanghai Key Laboratory of Medical Epigenetics International Co‐laboratory of Medical Epigenetics and Metabolism (Ministry of Science and Technology) Shanghai Cancer Center Fudan University Institutes of Biomedical Sciences Shanghai Medical College of Fudan University Shanghai China

**Keywords:** quantitative, RNA m6A, sequencing technology, single nucleotide

1

We recently reported m^6^A Selective Allyl Chemical labelling and sequencing (m^6^A‐SAC‐seq) to map whole‐transcriptome RNA m^6^A at single‐nucleotide resolution with stoichiometric information. This method does not rely on antibodies and can track the m^6^A dynamics with limited RNA samples. The m^6^A‐SAC‐Seq technology is currently the only method that can be widely used in various biological contexts and has good prospects in both basic biological research and clinical applications.

Based on the central dogma, genetic information is stored in DNA and mRNA is considered as message transmitter. The discovery of mRNA epigenetic modifications has expanded the function of mRNA. Post‐transcriptional modifications at the RNA level can regulate gene expression through cis‐ or trans‐mechanisms, which are crucial for cell function and cell fate determination. To date, more than 150 chemical modifications have been found on RNA, including m^6^A, m^1^A, pseudo‐uridine and m^5^C.^1^, ^2^ These modifications regulate gene expression at a critical level of the ‘epitranscriptome’.

m^6^A is the most abundant internal mRNA and the most extensively studied modification to date. Previous research work showed that m^6^A tends to occur in the (G/A) (m^6^A) C canonical motif. m^6^A affects the metabolic process of mRNA such as splicing, nuclear export, degradation and translation to regulate gene expression and plays important roles in developmental, physiological and pathological processes.^1^

The earliest whole‐transcriptome maps of m^6^A modifications were published in 2012 (m^6^A‐Seq or MeRIP‐Seq), using m^6^A antibodies to enrich m^6^A‐containing RNA fragments, resulting in profiling maps with the resolution of 100–200 nt.^3^, ^4^ The current mainstream sequencing method for RNA m^6^A is antibody‐based MeRIP. However, MeRIP has several shortcomings, including low resolution, inability to quantify, limitations in comparing m^6^A differences under different circumstances, and the need for a large amount of input RNA for library construction. m^6^A sequencing methods developed in recent years (such as Mazter‐seq,^5^ m^6^A‐REF‐seq,^6^ m^6^A‐label‐seq^7^ etc.) although greatly expand the dimension of m^6^A function research, have only partially solved the above‐mentioned limitations.

Recently, we published an article entitled ‘m^6^A RNA modifications are measured at single‐base resolution across the mammalian transcriptome’ in the journal *Nature Biotechnology*.^8^ The new method m^6^A‐SAC‐Seq (Selective Allyl Chemical labelling and sequencing) developed in this study starts to overcome this bottleneck. By directly labelling m^6^A, m^6^A‐SAC‐seq could recover m^6^A sites in Gm^6^AC canonical motifs, and detect m^6^A stoichiometry at single‐nucleotide resolution.

The strategy of m^6^A‐SAC‐seq is as follows (Figure 1): It utilizes the *Methanocaldococcus jannaschii* homolog MjDim1 from the Dim1/KsgA family of dimethyl transferases, which transfers the methyl group from S‐adenosyl‐L‐methionine (SAM) to adenosines, forming m^6^A and then N^6^, N^6^‐dimethyl‐adenosine (m^6^_2_A) in consecutive methylation reactions. MjDim1 shows highly processive kinetics of converting m^6^A into m^6^_2_A. Employing the chemically modified allylic‐SAM as the co‐factor, MjDim1 could label m^6^A with an allyl chemical group, converting m^6^A into allyl‐modified m^6^A (*N* ^6^‐allyl, *N* ^6^‐methyl adenosine or a^6^m^6^A). Subsequent I_2_ treatment converts a^6^m^6^A into homologs of N^1^, N^6^‐ethanoadenine.^9^ The HIV‐1 RT generates misincorporation at the cyclised a^6^m^6^A sites. m^6^A distribution in the transcriptome can be profiled based on the mutation site. Mutation rates of m^6^A in the calibration probes in different sequence contexts provide normalization standards to determine modification stoichiometries of individual m^6^A sites in sample RNA (Figure 2).

**FIGURE 1:**
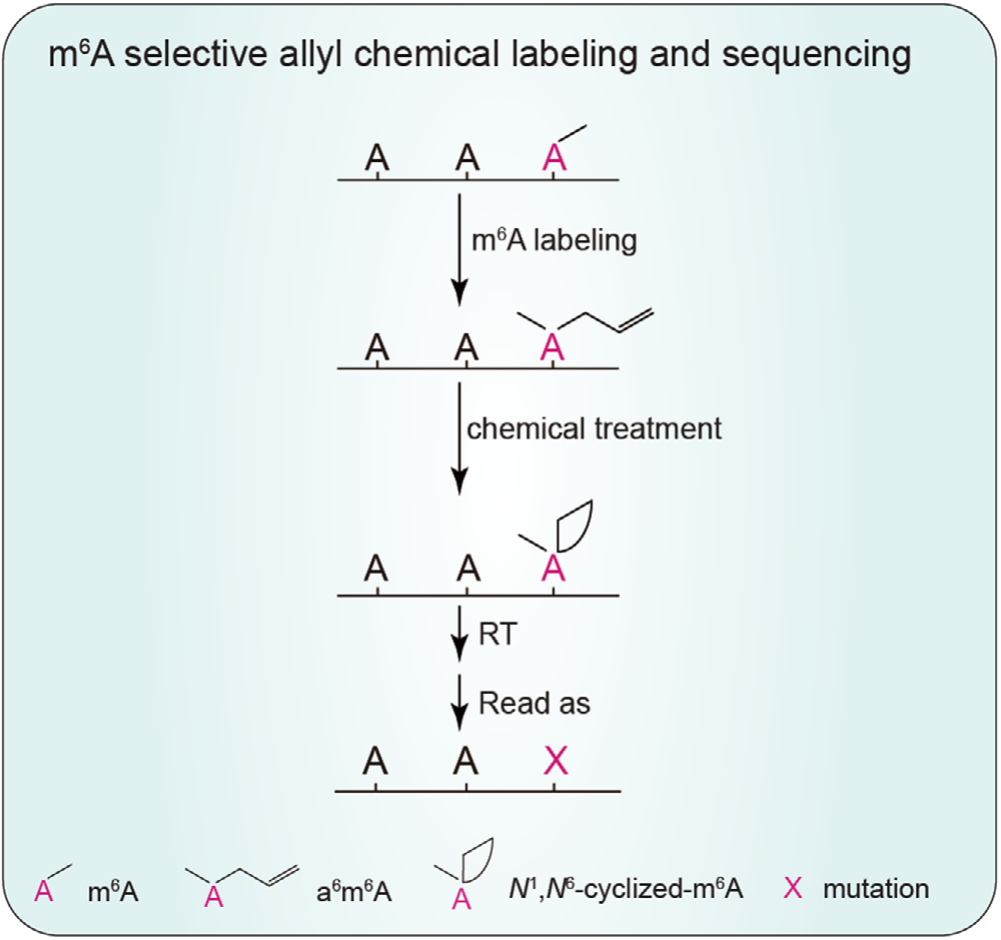
m^6^A‐SAC‐seq flow chart. m^6^A‐SAC‐seq utilises MjDim1 and allylic‐SAM as a co‐factor to label m^6^A to allyl^6^m^6^A, followed by cyclization upon I_2_ treatment. *N^1^, N^6^*‐cyclised‐m^6^A induce misincorporation during reverse transcription.

**FIGURE 2:**
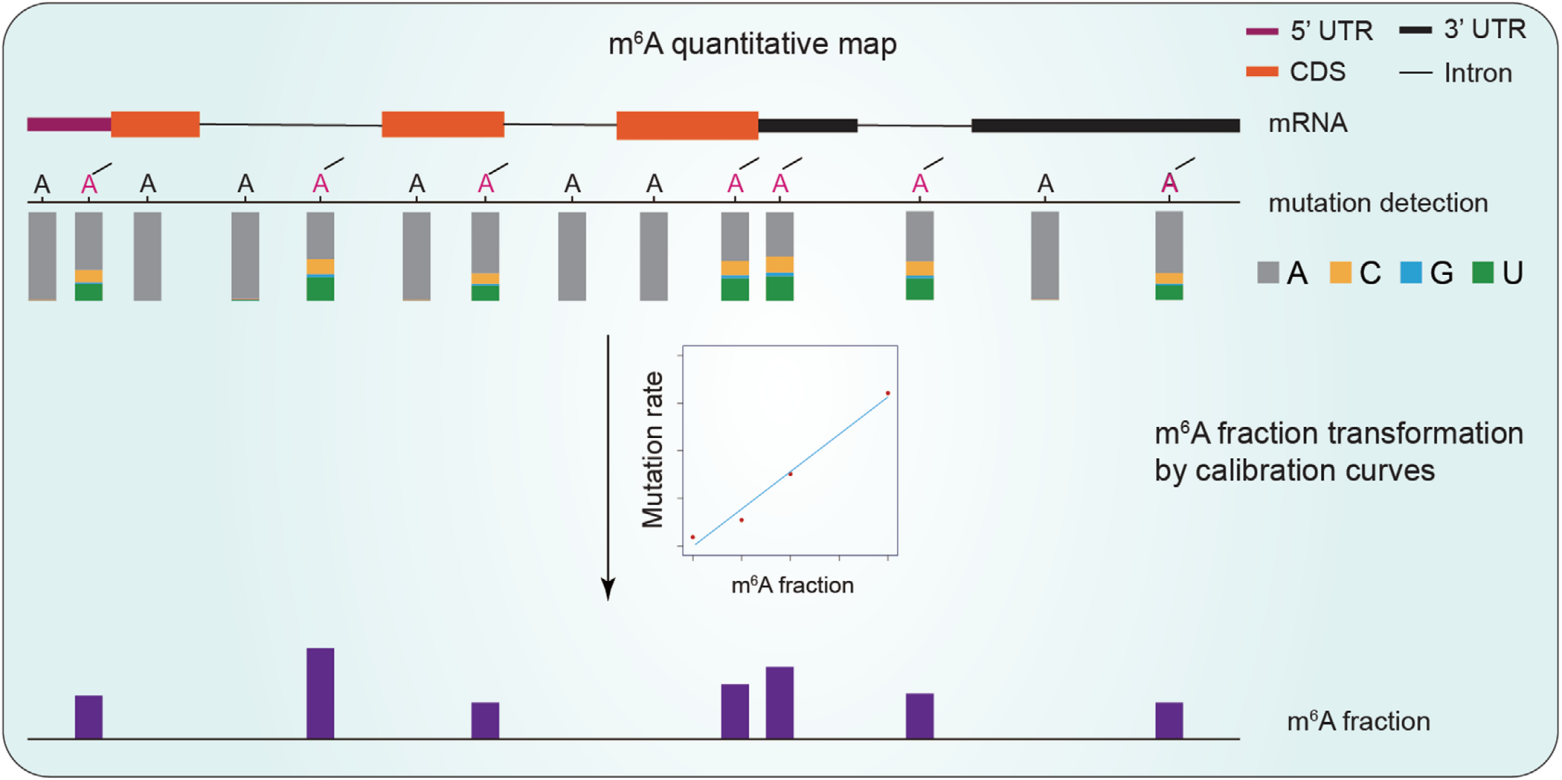
Calibration of the m^6^A stoichiometries with m^6^A‐SAC‐seq. Calibration curve for each GGACU motif is generated by linear regression. Mutation rates of m^6^A in the calibration probes in different sequence contexts provide normalization standards to determine RNA m^6^A modification stoichiometries.

Using m^6^A‐SAC‐seq, we identified approximately 10 000 loci with m^6^A stoichiometric information in each of HeLa, HEK293 and HepG2 cell lines, and the these m^6^A loci on transcripts were enriched at stop codons, CDS and 3′ UTR, consistent with previous reports. By combining the analysis of RNA degradation (decay) sequencing data, we also found that m^6^A stoichiometry and RNA half‐life showed significant negative correlation. Transcripts with high m^6^A stoichiometry tend to have short half‐life (lifetime). Our findings further clarify the function of m^6^A modification in the regulation of mRNA turnover.

In addition, we also successfully mapped the dynamics of m^6^A stoichiometry during human umbilical cord blood‐derived hematopoietic stem cells (HSPC) differentiation into monocytes using m^6^A‐SAC‐seq technology. During differentiation, m^6^A sites in different regions of the mRNA showed a large amount of redistribution and dynamic changes among the different regions of the mRNA during differentiation. This study demonstrates the potential of m^6^A‐SAC‐Seq technology to obtain transcriptome‐wide changes of m^6^A content, which could be informative to trace the m^6^A stoichiometric changes between normal and diseased tissue samples in clinical area.

Of note, although m^6^A‐SAC‐Seq technology requires 30 ng of mRNA or rRNA depleted total RNA in this study, we have subsequently optimized the method. The current experimental procedures only require 2 ng of mRNA or fragmented RNA extracted from FFPE sample. The m^6^A‐SAC‐Seq technology utilizes enzymatic labelling strategy to directly label m^6^A modification instead of harsh chemical treatment. Unlike MAZF strategy, it does not require reverse subtraction of unmodified ‘A’, hence greatly reducing background noise and improving the accuracy. The m^6^A‐SAC‐Seq technology has the technical advantage of low input RNA sample and is promising to be optimised to detect m^6^A at the single‐cell level.

The m^6^A‐SAC‐Seq technology has great potential to become a ‘gold standard’ that conquers the technological bottleneck of quantitative m^6^A sequencing and boosts clinical research based on tissue and cell free RNA modification.

## CONFLICT OF INTEREST

A patent application for m^6^A‐SAC‐seq has been filed by the University of Chicago.

## References

[1] Frye M , Harada BT , Behm M , He C . RNA modifications modulate gene expression during development. Science. 2018;361:1346‐1349. 10.1126/science.aau1646 30262497 PMC6436390

[2] Roundtree IA , Evans ME , Pan T , He C . Dynamic RNA modifications in gene expression regulation. Cell. 2017;169:1187‐1200. 10.1016/j.cell.2017.05.045 28622506 PMC5657247

[3] Dominissini D , Moshitch‐Moshkovitz S , Schwartz S , et al. Topology of the human and mouse m6A RNA methylomes revealed by m6A‐seq. Nature. 2012;485:201‐206. 10.1038/nature11112 22575960

[4] Meyer KD , Saletore Y , Zumbo P , Elemento O , Mason CE , Jaffrey SR . Comprehensive analysis of mRNA methylation reveals enrichment in 3' UTRs and near stop codons. Cell. 2012;149:1635‐1646. 10.1016/j.cell.2012.05.003 22608085 PMC3383396

[5] Garcia‐Campos MA , Edelheit S , Toth U , et al. Deciphering the “m(6)A Code” via antibody‐independent quantitative profiling. Cell. 2019;178:731‐747.e16. 10.1016/j.cell.2019.06.013. e716. 31257032

[6] Zhang Z , Chen Li‐Q , Zhao Yu‐Li , et al. Single‐base mapping of m(6)A by an antibody‐independent method. Sci Adv. 2019;5:eaax0250. 10.1126/sciadv.aax0250 31281898 PMC6609220

[7] Shu X , Cao J , Cheng M , et al. A metabolic labeling method detects m(6)A transcriptome‐wide at single base resolution. Nat Chem Biol. 2020;16:887‐895. 10.1038/s41589-020-0526-9 32341503

[8] Hu L , Liu S , Peng Y , et al. m(6)A RNA modifications are measured at single‐base resolution across the mammalian transcriptome. Nat Biotechnol. 2022:1210‐1219. 10.1038/s41587-022-01243-z 35288668 PMC9378555

[9] Shu X , Dai Q , Wu T , et al. N(6)‐Allyladenosine: a new small molecule for RNA labeling identified by mutation assay. J Am Chem Soc. 2017;139:17213‐17216. 10.1021/jacs.7b06837 29116772 PMC5813804

